# Role of the DSC1 Channel in Regulating Neuronal Excitability in *Drosophila melanogaster*: Extending Nervous System Stability under Stress

**DOI:** 10.1371/journal.pgen.1003327

**Published:** 2013-03-07

**Authors:** Tianxiang Zhang, Zhe Wang, Lingxin Wang, Ningguang Luo, Lan Jiang, Zhiqi Liu, Chun-Fang Wu, Ke Dong

**Affiliations:** 1 Department of Entomology, Michigan State University, East Lansing, Michigan, United States of America; 2 Department of Microbiology and Molecular Genetdics, Michigan State University, East Lansing, Michigan, United States of America; 3 Department of Biology, University of Iowa, Iowa City, Iowa, United States of America; 4 Department of Biological Sciences, Oakland University, Rochester, Michigan, United States of America; North Carolina State University, United States of America

## Abstract

Voltage-gated ion channels are essential for electrical signaling in neurons and other excitable cells. Among them, voltage-gated sodium and calcium channels are four-domain proteins, and ion selectivity is strongly influenced by a ring of amino acids in the pore regions of these channels. Sodium channels contain a DEKA motif (i.e., amino acids D, E, K, and A at the pore positions of domains I, II, III, and IV, respectively), whereas voltage-gated calcium channels contain an EEEE motif (i.e., acidic residues, E, at all four positions). Recently, a novel family of ion channel proteins that contain an intermediate DEEA motif has been found in a variety of invertebrate species. However, the physiological role of this new family of ion channels in animal biology remains elusive. DSC1 in *Drosophila melanogaster* is a prototype of this new family of ion channels. In this study, we generated two *DSC1* knockout lines using ends-out gene targeting via homologous recombination. *DSC1* mutant flies exhibited impaired olfaction and a distinct jumpy phenotype that is intensified by heat shock and starvation. Electrophysiological analysis of the giant fiber system (GFS), a well-defined central neural circuit, revealed that *DSC1* mutants are altered in the activities of the GFS, including the ability of the GFS to follow repetitive stimulation (i.e., following ability) and response to heat shock, starvation, and pyrethroid insecticides. These results reveal an important role of the DSC1 channel in modulating the stability of neural circuits, particularly under environmental stresses, likely by maintaining the sustainability of synaptic transmission.

## Author Summary

Voltage-gated sodium and calcium channels are four-domain proteins that are essential for electrical signaling in neurons and other excitable cells. Recent genomic and functional analyses reveal a novel family of four-domain, Ca^2+^-selective cation channels in a variety of invertebrates, from sea anemones to insects. The amino acid sequences and gating properties of these channels appear to be intermediate between sodium and calcium channels; as such, these channels could potentially be an important evolutionary link between sodium and calcium channels. Despite the intriguing nature of this family of cation channels, their role in animal physiology remains mysterious. In this study, taking advantage of the genetic tractability of the fruit fly, *Drosophila melanogaster*, we examined the physiological role of such a channel, called DSC1, in this model insect. We generated two *DSC1* knockout lines and conducted behavioral and electrophysiological analyses. Our results show that the DSC1 channel contributes to neuronal excitability regulation and plays a unique role in retaining stability of the nervous system function in response to environmental stresses, including heat shock and starvation. Interestingly, the *DSC1* knockout flies were also more susceptible to pyrethroid insecticides, which are used globally as a major weapon against the malaria-carrying mosquitoes.

## Introduction

Voltage-gated sodium channels are primarily responsible for the initiation and propagation of action potentials, playing a critical role in regulating neuronal excitability. They are members of a superfamily that also includes voltage-gated potassium channels and voltage gated calcium channel [1]. The sodium and calcium channels contain four homologous domains, whereas the potassium channels consist of tetramers of single-domain subunits. It is generally believed that sodium channels evolved from an ancient calcium channel [2], [3]. Selectivity in sodium and calcium channels is strongly influenced by a ring of amino acids in the pore regions of the channels [4]. Sodium channels contain a DEKA motif (i.e., amino acids D, E, K, and A at the pore positions of domains I, II, III, and IV, respectively), whereas voltage-gated calcium channels contain an EEEE motif (i.e., acidic residues, E, at all four positions).

Recently, a novel family of ion channel proteins that contains an intermediate DEEA motif in the pore regions has been found in a variety of invertebrate species [5]. DSC1 in *Drosophila melanogaster* and BSC1 in *Blattella germanica*, are prototypes of this new family of ion channels. We have previously shown that DSC1 and BSC1 are functionally and evolutionally intermediate between voltage-gated sodium and calcium channels and more permeable to Ca^2+^ even though the amino acid sequences are more closely related to sodium channels [6], [7]. Interestingly, a recent study showed that a sodium channel-like channel containing a DEEA motif, NvNa_v_2.1, from the starlet sea anemone also conducts Ca^2+^ [5]. Therefore, it has been speculated that the DEEA motif could be a pore sequence from which sodium channels have evolved from calcium channels [2], [5]. More strikingly, a recent phylogenetic analysis revealed the presence of the DEEA motif in a gene homologous to voltage-gated sodium channels in a single-celled choanoflagellate, indicating that evolution of sodium channels may have predated the origin of the nervous systems [2]. Intriguingly, this DEEA motif was retained in many other animal groups, such as ascidians, insects and cnidarians, but was lost in vertebrates [2], [5]. The physiological role of this new family of sodium channel-like, but Ca^2+^-selective, ion channels in animal biology, however, remains mysterious.

In this study, we investigate the role of the DSC1 channel *in vivo*. The *DSC1* transcript and the DSC1 protein are found in a variety of tissues, such as brain, antennae, thorax, legs, and ovary [8], [9], suggesting a potentially broad role of DSC1 in insect biology. Previously, Anholt and colleagues conducted genetic and molecular analysis of a *smell-impaired* (*smi*) mutant, *smi60E*, which carried a *P*-element insertion in an intron of the *DSC1* gene [10]. This insertion resulted in a 2-fold reduction in the steady-state level of the *DSC1* transcript and the mutant had a slight reduction in olfactory response to benzaldehyde, implicating a role of the DSC1 channel in olfaction [10]. However, a complete knockout mutant of *DSC1* has not been characterized, and it remains to be determined whether DSC1 has a broader role in modulating insect neurophysiology.

We generated two *DSC1* knockout lines and conducted a battery of behavioral analyses, followed by electrophysiological characterization of the giant fiber system (GFS), a well-defined central neural circuit [11], [12], [13], [14]. Our results not only confirm a role of the DSC1 channel in insect olfaction, but, unexpectedly, also show that the DSC1 channel has a unique role in regulating neuronal excitability, especially in extending the stability of neural circuits and behaviors under environmental stresses and insecticide exposure.

## Results

### Targeted knockout of the *DSC1* gene by homologous recombination

**Figure 1:**
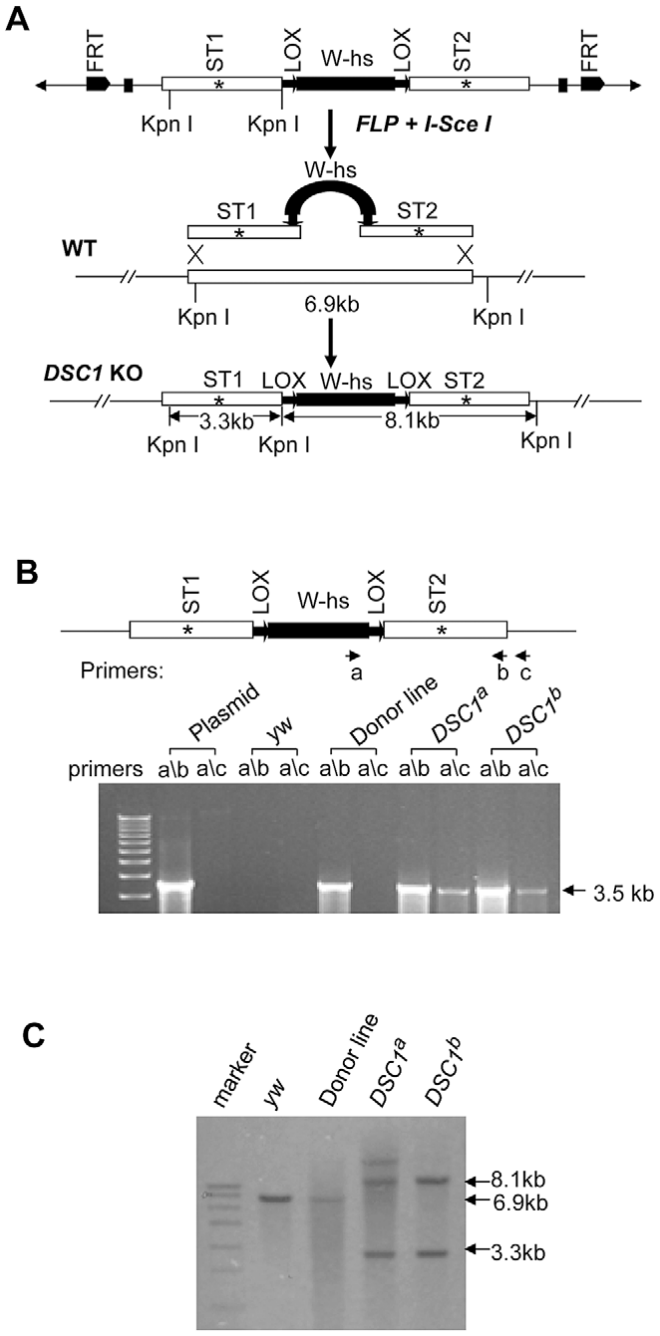
Method for targeted gene knockout and confirmation by PCR and Southern blot analysis. (*A*) Schematic presentation of the donor construct, homologous recombination and replacement of the endogenous *DSC1* sequence with the donor sequences carrying stop codons (_*_). A 6.6-kb *DSC1* genomic DNA region was amplified in two 3.3-kb fragments and a stop codon was introduced into the middle of each fragment (i.e., ST1 in the upstream fragment and ST2 in the downstream fragment). The upstream and downstream fragments were then cloned into the *pW25* vector (shown at the top in *A*). The donor construct was transformed into *w^1118^* flies to generate donor construct lines. The donor construct lines were crossed with another transgenic line that contains heat-inducible *70FLP* recombinase and *70I-Sce*I endonuclease genes to induce *DSC1*-targeted homologous recombination. (*B*) Confirmation of *DSC1* knockout by genomic PCR. Amplification of a 3.6 kb *DSC1* genomic fragment using the primer pairs a/c from *DSC1* knockout flies, but not from donor flies. (*C*) Southern blot analysis. Genomic DNA from WT (lane 2), a donor construct line (lane 3), and two independent homozygous *DSC1* knockout lines (lanes 4 and 5) was digested with the restriction enzyme *KpnI* and hybridized with two genomic DNA probes made from the two 3.3-kb *DSC1* fragments (in *A*). As expected, the wild-type 6.9-kb band was converted into two bands of 3.3 kb and 8.1 kb in the *DSC1* knockout lines due to homologous recombination.

Although a *P*-element insertion has been found in an intron of the *DSC1* gene in the *smi60E* mutant, which has partially reduced expression of the *DSC1* transcript level and decreased olfactory response to benzaldehyde [10], complete knockout mutants of the *DSC1* gene would be ideal for assessing the full extent by which the *DSC1* gene contributes to insect biology. We successfully used the method of ends-out targeted gene knockout via homologous recombination [15] to isolate two independent *DSC1* knockout lines. The procedures for generating *DSC1* knockout lines are summarized in Figure 1A. Twelve independent transgenic lines were mapped to the chromosome 2 (where the *DSC1* gene resides). To detect the targeting events in *DSC1* knockout flies by PCR, two pairs of primers were used. Primer a corresponds to the inserted *white* gene, and primers b and c to the *DSC1* genomic DNA sequences inside and outside of the donor construct (Figure 1B). As expected, there was no detectable PCR product amplified from control flies using primer pairs a/b or a/c. A PCR product with the predicted length (3.5 kb) was amplified from *DSC1* transgenic donor flies using primer pair a/b but not a/c. On the other hand, predicted PCR products of 3.5 kb and 3.6 kb were amplified from two targeted transgenic lines using primers a/b and a/c, respectively (Figure 1B). These two lines are named *DSC1^a^* and *DSC1^b^*.

Southern blot analysis was also performed to confirm *DSC1* knock-out. DNA from homozygous flies was digested with restriction enzyme *KpnI* and hybridized with a probe made from the *DSC1* fragments (Figure 1C). As predicted, a 6.9 kb band in the wild-type and two bands of 3.3 kb and 8.1 kb in *DSC1^a^* and *DSC1^b^* flies were detected (Figure 1C).

### Behavioral characterization of *DSC1* knockout flies

The successful construction of two independent *DSC1* knockout lines provided us with a critical foundation for characterizing the role of the DSC1 channel in insect biology. Previous research has shown a broad distribution of the *DSC1* transcript and the DSC1 protein in different tissues, such as brain, antennae, thorax, legs, and ovary [8], [9], yet, the *DSC1* knockout flies are viable under the standard laboratory rearing condition and exhibit no morphological and developmental abnormalities and have a normal lifespan and weight, which enabled us to perform a variety of behavioral tests to assess *in vivo* function of the DSC1 channel in *D. melanogaster*.

Because the DSC1 channel was implicated in olfaction in a previous study [10], we first conducted an olfaction behavioral assay. As expected, the defect in olfaction was more severe in the *DSC1^a^* and *DSC1^b^* flies than that previously observed in the *smi60E* mutant (Figure S1A and S1B).

**Figure 2:**
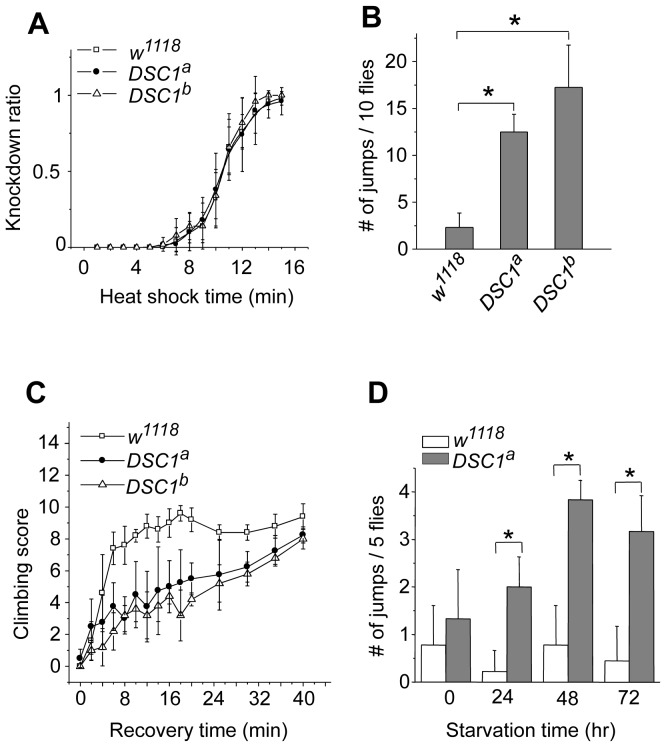
Behavioral characterization of *DSC1* knockout mutants. (*A*) Knockdown time courses for *w^1118^* and two independent *DSC1* knockout flies after incubation at 40°C for various times up to 15 minutes (*n* = 50 for each line). (*B*) The number of jumps observed during the fifth to tenth minute of incubation at 40°C with no mechanical disturbance (*n* = 50 for each line). (*C*) Recovery of *w^1118^* and *DSC1* knockout flies from the heat shock (*n* = 50 for each line) (*D*) The number of jumps of starved flies upon mechanical disturbance. The number of jumps per vial (five flies) was recorded during the first second to the tenth seconds after a gentle tap on the vial (*n* = 50 for each line). Data are presented as mean and standard deviation (* *p*<0.05, Student's *t*-test).

Next, we performed experiments to determine possible locomotion defects in the *DSC1* knockout mutants. Climbing assays were carried out as described in Protocol S1. After 30 sec, more than 80% of *DSC1^a^* and *DSC1^b^* flies were able to reach or pass the 10 cm bar and climbed into the second vial, with no significant difference from that of the *w^1118^* control flies (Figure S1C). However, *DSC1^a^* and *DSC1^b^* flies had a stronger tendency to jump or fly when disturbed by a gentle tap on the vial (see Video S1). The jumpy phenotype suggests a defect in the nervous system in the *DSC1* knockout mutants since the DSC1 protein is not detected in muscles [8]. We noticed that this defect was especially evident during experiments to assess the response of the *DSC1* knockout mutants to heat shock and starvation. Unlike other *Drosophila* ion channel mutants, DSC1 KO flies did not exhibit leg-shaking under ether anesthesia or temperature-sensitive paralysis. Both *w^1118^* and mutant flies began to exhibit knockdown (unable to walk or on their back) 5 min into 40°C heat shock and all flies were knocked down at the end of 15 min heat shock (Figure 2A). However, *DSC1* knockout flies were significantly more jumpy (frequently flying off from the wall or bottom of the vial) than *w^1118^* flies, particularly from the fifth to tenth minutes after heat shock (Figure 2B). Furthermore, the recovery from heat shock was significantly delayed in *DSC1* knockout flies when the vials were returned to room temperature (Figure 2C). At the 10-min time point, 80% of *w^1118^* flies and 40% of *DSC1* knockout flies had resumed climbing. However, it took an additional 30 min for another 40% of *DSC1* knockout flies to climb up again (Figure 2C). Similarly, in experiments to determine the response of *DSC1* knockout flies to starvation, *DSC1^a^* flies were significantly more jumpy than *w^1118^* flies after starvation (Figure 2D; see Video S2).

### *DSC1* knockout flies are more susceptible to pyrethroids, but not to DCJW and fipronil

The DSC1 channel shares the highest sequence similarity with the Para sodium channel which is the primary target of pyrethroid insecticides and sodium channel blocker insecticides (SCBI). We first attempted to determine whether the DSC1 channel is directly affected by these sodium channel-targeting insecticides by pharmacologically characterizing DSC1 channels expressed in *Xenopus* oocytes. However, DSC1 currents in oocytes exhibit significant rundown, which makes pharmacological analysis unfeasible.

We therefore took an alternative approach to assess the involvement of DSC1 in possibly modulating pyrethroid sensitivity. Pyrethroids prolong the opening of sodium channels, causing increased neuronal excitability including repetitive firing and/or membrane depolarization (depending on doses of pyrethroid, types of pyrethroids, and also nerve preparations) [16]. Sustained action of pyrethroids eventually results in the complete blockage of signal transmission and lethality of poisoned insects [16]. We examined the susceptibility of *DSC1* knockout flies to the lethal effect of pyrethroids. Flies from *w^1118^* and both *DSC1* knockout lines, *DSC1^a^* and *DSC1^b^*, were tested in a contact bioassay. We found that *DSC1* knockout mutants were more susceptible than *w^1118^* flies to all four pyrethroids tested, including two type I (permethrin and bioresmethrin) and two type II (deltamethrin and fenvalerate) pyrethroids (Table 1, Table S2).

**Table 1:** Susceptibility of *w^1118^* and *DSC1* knockout flies to insecticides.

	LC_50_ 1 (µg/vial)								
Insecticide	*w^1118^* (95% CI^2^)	*n* 3	Slope (SE)	*DSC1^a^* (95% CI)	*n*	Slope (SE)	*DSC1^b^* (95% CI)	*n*	Slope (SE)
permethrin	14.0 (11.6–16.6)	1680	3.47 (0.23)	5.0 (4.4–5.8)*	700	3.06 (0.25)	5.4 (4.2–6.7)*	600	3.95 (0.34)
bioresemethrin	3.8 (3.3–4.3)	640	2.68 (0.21)	2.2 (2.0–2.4)*	640	3.32 (0.27)	2.0 (1.8–2.3)*	640	3.41 (0.27)
deltamethrin	0.5 (0.4–0.6)*	2220	2.79 (0.14)	0.3 (0.2–0.3)*	640	1.96 (0.15)	0.2 (0.2–0.3)*	240	3.43 (0.62)
fenvalerate	3.1 (2.7–3.4)*	860	3.28 (0.32)	1.4 (0.9–1.9)*	1160	2.5 (0.23)	1.7 (1.0–2.4)*	860	2.15 (0.21)
DCJW	2.1 (1.7–2.5)	720	1.48 (0.15)	1.9 (1.5–2.3)	720	1.43 (0.15)	ND^4^		ND
fipronil	1.6 (0.7–5.5)	420	1.58 (0.15)	1.1 (0.8–1.9)	480	1.74 (0.16)	ND		ND

1 LC_50_ : concentration required to kill 50%.

2 CI: confidence interval.

3 *n*: number of flies tested.

4 ND: Not determined.

* CI not overlapped with that of *w^1118^*.

Next, we examined whether the hyper-susceptibility of the *DSC1* knockout mutant is specific to pyrethroids. We tested DCJW, an active metabolite of indoxacarb, which is a sodium channel blocker insecticide (SCBI) and has a mode of action opposite to pyrethroids (i.e., inhibiting neuronal excitability) [17]. As shown in Table 1, the susceptibilities of *DSC1* knockout flies to DCJW were similar to that of *w^1118^* flies. To determine whether the DSC1 channel may have effects on susceptibility beyond sodium channel-targeting insecticides, we tested the susceptibility of *DSC1* flies to the insecticide fipronil, which causes neuronal hyperexcitability by blocking the GABA-gated Cl^−^ channel [18]. As shown in Table 1, *w^1118^* and *DSC1* knockout flies exhibited similar susceptibility to fipronil. These results suggest that *DSC1* knockout flies are affected in response to neuronal stimulation by sodium channel activators.

### DSC1 modulates the giant fiber system-mediated long-latency refractory period in the dorsal longitudinal muscles (DLM) branch

**Figure 3:**
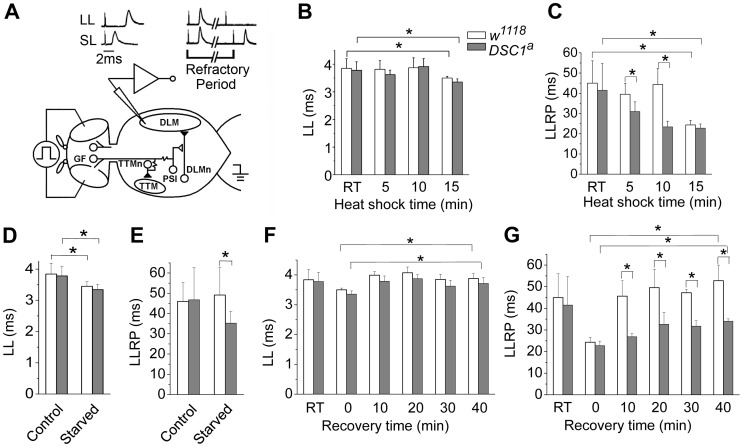
The GFS activities of *w^1118^* and *DSC^1^* knockout flies under heat shock and starvation. (*A*) The giant fiber system (GFS) diagram. A bilateral pair of the GF neurons in the brain sends out a descending pair of giant axons into the ventral thoracic ganglion and makes mixed electric-chemical synapses with the motorneuron of the tergotrochanteral muscle (TTM, the jump muscle) and an interneuron, the peripherally synapsing interneuron (PSI). The PSI in turn makes a cholinergic synapse with the motorneurons of the dorsal longitudinal muscles (DLMs), which power flight. Two stimulating electrodes are inserted in the left and right compound eyes to deliver electric stimuli across the brain. A ground electrode is inserted into the abdomen. Activation of the motorneurons produces the characteristic spikes in the TTM and the DLM, which are recorded as the GFS output. (*B–G*) Giant fiber recording of the latency and the refractory period of the long latency response of *w^1118^* and *DSC1^a^* flies at different time points during heat shock (*B and C*), starvation (*D and E*), and recovery from heat shock (*F* and *G*). The latencies and refractory periods measured at room temperature (RT) before heat shock were included for comparison. LL, long latency; LLRP, long latency refractory period. Data are presented as mean and standard deviation (* *p*<0.05, Two-way ANOVA).

To identify potential defects in the nervous system of *DSC1* knockout flies, we examined a well-defined adult neural circuit, the giant fiber system (GFS; Figure 3A). The GFS mediates the jump-and-flight escape reflex in response to visual stimuli in *Drosophila*. The components of the GFS are depicted in Figure 3, including the tergotrochanteral muscle (TTM) for jump and the dorsal longitudinal muscle (DLM) for flight. Briefly, the somas of giant fiber neurons are located in the brain with their large axons extending to the thorax, where the terminals of the giant fiber axon form synaptic connections with two different neurons: a large motorneuron that innervates the TTM and a peripherally synapsing interneuron (PSI). The PSI axon crosses the midline and synapses with motor neurons, which innervate the DLM. The giant fiber pathway can be triggered from different sites (the brain or thorax), recruited at different stimulation intensities. The responses can be recorded from DLM or TTM which represent two distinct branches: the GF-PSI-DLMn-DLM and GF-TTMn-TTM (see Figure 3 legend for details). The time interval between the stimulus and the first muscle potential is termed the response latency. It reflects the chain of events from stimulation, initiation and conduction of action potentials, and synaptic transmission along the neuronal elements of the neural circuit to the innervated muscle. At higher stimulating voltages, which are sufficient to directly excite the giant fiber, muscle spikes can be recorded after a shorter time, defining a short-latency (SL) response (Figure 3A). When lower stimulating voltages are used, the same set of muscle potentials appears but only after a longer delay because lower-intensity stimuli across the brain recruit the upstream synaptic activity afferent to the GF and thus trigger the muscle response with a longer delay (Figure 3A). Thus, high and low voltage stimuli result in SL or long-latency (LL) responses, respectively.

The refractory period (RP) is the characteristic time interval following the first stimulus during which a second stimulus fails to evoke another response. We found that the LL and SL responses from the DLM branch and their corresponding refractory periods were similar between undisturbed *w^1118^* and *DSC1* knockout flies (Figure 3 and Figure S2, Table S3). However, the LL and SL responses and their refractory periods of flies under heat shock were significantly different. The LL response and the long-latency refractory period (LLRP) were decreased upon heat shock in both *w^1118^* and *DSC1* knockout flies (Figure 3B and 3C, Table S3). Notably, the reduction in LLRP was significantly more drastic in *DSC1* knockout flies at 5 and 10 minutes after heat shock, coinciding with the time when the jumpy phenotype in *DSC1* knockout mutants was most pronounced (Figure 2B). At the end of the 15 min heat shock, the LLRP of *w^1118^* flies also was drastically reduced, but no difference was observed between *w^1118^* and *DSC1* mutant flies, consistent with the eventual total paralysis of both *w^1118^* and *DSC1* knockout flies. In contrast to the LLRP results, *w^1118^* and *DSC1* knockout flies showed similar SL refractory periods (SLRP) (Figure S2). No difference in the latency of the LL response was observed between *w^1118^* and *DSC1* mutant flies in response to starvation (Figure 3D, Table S4). However, an apparently LLRP-specific defect was detected in *DSC1* mutant flies when starved flies were assayed (Figure 3E, Table S4), consistent with the exaggerated jumpy phenotype of *DSC1* knockout flies under starvation (Figure 2D).

Because the recovery from heat shock was delayed for the *DSC1* knockout flies compared to *w^1118^* flies (Figure 2C), we examined the changes in GFS parameters during the recovery at 10, 20, 30 and 40 min. The reduced LL was recovered in both *w^1118^* and *DSC1* knockout flies within 10 minutes (Figure 3F, Table S5). Again, we detected a significant difference in the LLRP between *w^1118^* and the *DSC1* knockout flies (Figure 3G). Whereas the LLRP was fully recovered in *w^1118^* flies at 10 min, it was still significantly shorter at the end of 40 min for *DSC1* knockout flies (Figure 3G, Table S5).

### The ability of the GFS to follow high frequency stimulation is impaired in *DSC1* knockout flies

**Figure 4:**
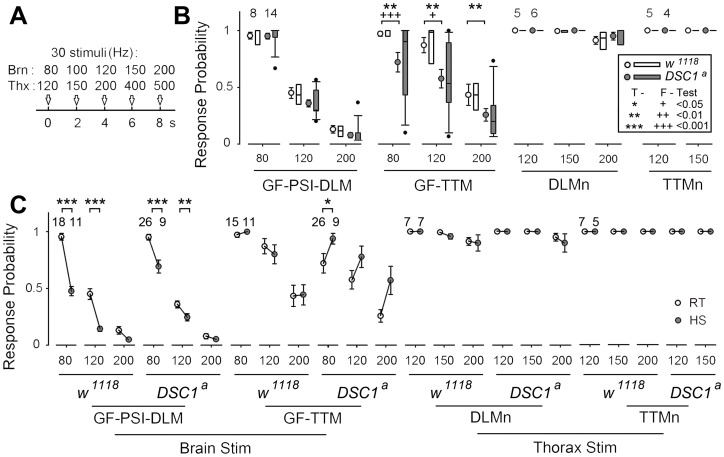
Localization of altered responses to high-frequency stimulation in the DLM and TTM branches in *DSC1* knockout flies at room temperature and following heat stress. (*A*) The stimulation protocols for measuring the frequency responses from different segments of the GF pathway are illustrated. Briefly, 5 trains of 30 pulses of various frequencies (indicated) were delivered sequentially via brain (Brn) or thorax (Thx) stimulation and recordings were made from both DLM and TTM. The stimulus intensity was set at 1.5× the threshold of SL responses (for brain stimulation) or 1.5× the threshold of DLMn responses (for thorax stimulation) to ensure the stimulus reliably recruits target neurons/pathways to trigger TTM and DLM responses. (*B*) Responses from the GF-PSI-DLM and the GF-TTM branches (left panels, brain stimulation) and from the DLMn and TTMn (right panels, thorax stimulation). In each group, mean and standard error of the mean are presented on the left and distribution of the data points is presented as box plots on the right. Sample sizes (number of flies) for each experimental group are shown. Statistically significant differences in sample means (*t*-Tests) and variances (F-Tests) are also indicated (* and +, see inset legend). Note that it is technically difficult to distinguish smaller TTM spikes above 200 Hz due to excessive masking of artifacts and muscle contraction. Therefore, no data above 150 Hz was presented for TTMn. (C) Effects of heat shock on high-frequency stimulation responses of various segments of the GFS. Responses before (same as in B) and after 15 min heat shock are compared for different frequencies of brain and thorax stimulation. The response means and standard errors for the room-temperature (RT, open circles) and heat- shock (HS, shaded circles) groups are paired and linked.

The GFS is extremely robust to high frequency stimulation [11], [19]. The greater reduction in the LLRP of *DSC1* knockout flies in response to heat shock and starvation suggests these treatments destabilize the function of the GFS. Thus, we applied different stimulus protocols to localize the weaker links along the GF pathway as revealed by altered responses to repetitive stimulation. We challenged the GF-PSI-DLM and GF-TTM branches of the GFS with 5 trains of 30 stimuli of different frequencies, up to 200 Hz (Figure 4A) and recorded the SL responses from the DLM and TTM (Figure 4B). Notably, unlike DLM, it is evident that the response from the TTM was significantly impaired in *DSC1* knockout flies as compared to *w^1118^* flies (Figure 4B). Besides the drop in the overall mean frequency response, the frequency response of the GF-TTM branch in *DSC1* knockout flies is also highly variable, which could undermine the stability or reliability of the motor output. The response in the DLM branch upon brain stimulation, however, was not different between *w^1118^* and *DSC1* knockout flies (Figure 4B), which could be due to the fact that the peripherally synapsing interneuron (PSI), a cholinergic neuron interposing between the GF and DLMn, is an intrinsically weaker point in high frequency response [20], which masks any defect in the GF itself in *DSC1* flies occurring at higher frequencies. Since the TTM branch does not involve the PSI, we could observe a defect in the following ability of the TTM branch. These findings demonstrate that the GF itself in mutant flies harbors electric signaling defects, suggesting an important role of the DSC1 channel in maintaining stable high-frequency signal transmission along the GF circuit.

To determine whether the GF-TTM impairment exists beyond the GF at the motorneuron level (see Figure 3A for circuit), we applied thorax stimuli to bypass the GF and directly recruit DLMn and TTMn. We found that the responses of DLM and TTM were similar between *w^1118^* and *DSC1* knockout flies (Figure 4B), indicating that the following abilities of both the DLMn and TTMn were similar and well above those of the GF-TTM branch. Therefore, the primary defect can be located to the GF itself.

We then examined the effects of heat shock on following abilities of the GF-TTM and GF-PSI-DLM branches. A significant decrease in frequency response was observed in the GF-PSI-DLMn branch in both *w^1118^* and *DSC1* knockout flies, presumably due to further weakening of PSI frequency response by high temperature (Figure 4C). In contrast, the GF-TTM response was not obviously changed in *w^1118^* flies but a tendency of enhanced frequency response was observed in *DSC1* knockout flies, a potential indication of heightened excitability related to the heat shock-induced “jumpy” phenotype of the mutant flies. Furthermore, heat shock did not significantly alter the frequency response of the downstream motor neurons, TTMn and DLMn, in both *w^1118^* and *DSC1* knockout flies (Figure 4C). Thus, the results demonstrate that different neuronal elements in the GF circuit are differentially affected in *DSC1* knockout flies and that high temperature treatment could lead to responses very different from that in control flies. In particular, the central GF-TTMn transmission in the *DSC1* knockout flies displayed severe defect and instability, as well as drastically different heat shock response, whereas the ability of TTMn or DLMn to drive the TTM and DLM via peripheral synapses remains largely intact in *DSC1* knockout flies even after heat shock (Figure 4C).

### The DLM branch of *DSC1* knockout flies is more sensitive to pyrethroids

**Figure 5:**
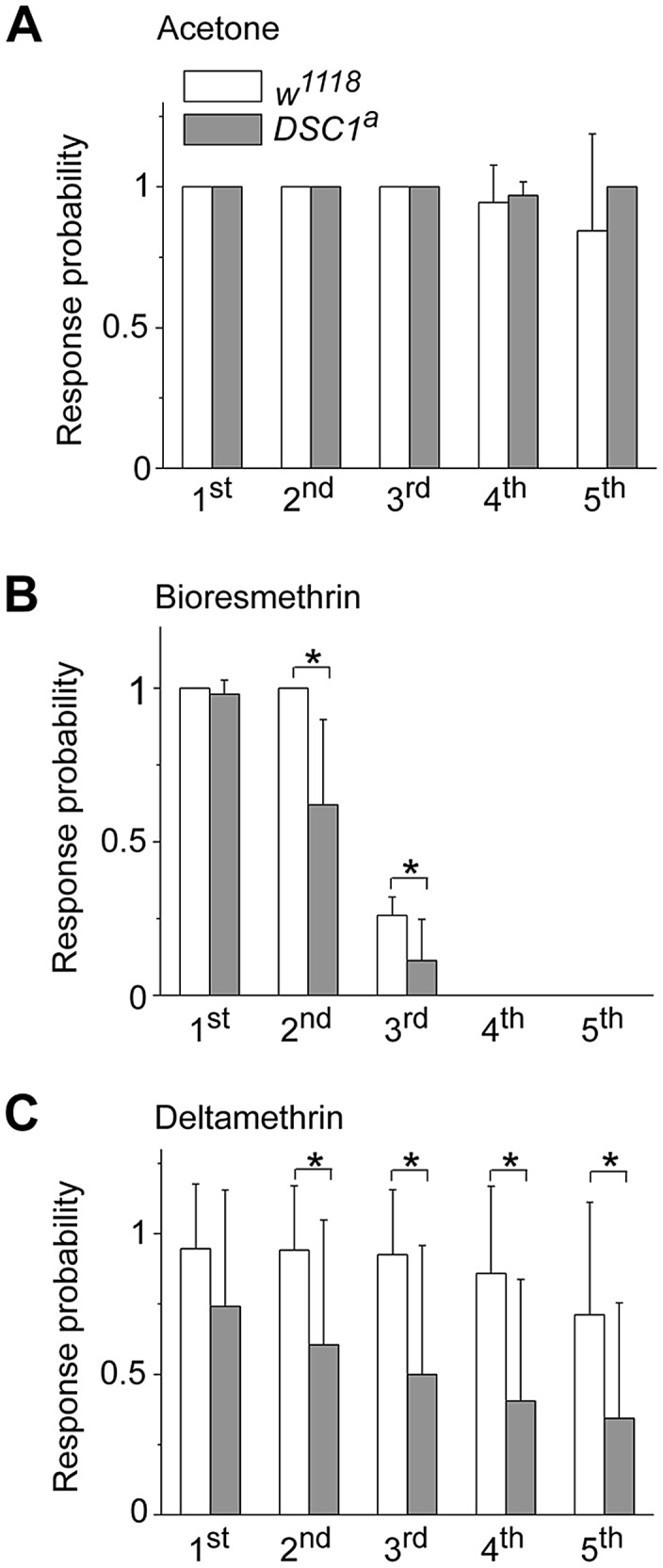
Comparisons of the responses to high-frequency stimulation in the DLM branch between *w^1118^* and *DSC1* knockout flies after pyrethroid exposure. Number of SL responses of DLMs triggered by each group of 10 stimuli at 100 Hz was recorded after 15-minute exposure to acetone (*A*), 0.4 ng/0.2 µl deltamethrin (*B*) or 4 ng/0.2 µl bioresmethrin (*C*); and normalized to that in control. The amplitude and duration of each pulse were 30 volts and 0.1 ms, respectively. Pyrethroids were topically delivered onto the dorsal side of thorax. Data are presented as mean and standard deviation (* *p*<0.05, Student's *t*-test).

To determine whether the enhanced sensitivity to pyrethroids may also be linked to an altered sensitivity of the GFS in *DSC1* knockout flies, we examined the effect of pyrethroids on the activities of the DLM branch since the ability of the DLM branch to follow high frequency stimulation was not altered in *DSC1* knockout flies (Figure 4B). The flies were examined fifteen minutes after exposure to pyrethroids by topical application to the dorsal thorax (See Protocol S1). In response to a train of 50 pulses delivered at 100 Hz and 30 V, each stimulus evoked a muscle potential from the DLM and a total of 50 muscle potentials were observed in both *w^1118^* and *DSC1^a^* knockout flies (Figure 5A), which is consistent with the results in Figure 4B. The responses, however, were altered after pyrethroid exposure. In *w^1118^* flies, the number of muscle potentials during the third 10 stimuli was reduced by bioresmethrin; and no muscle potentials were elicited in the remaining two 10 stimuli (Figure 5B). *DSC1^a^* flies were more sensitive to bioresmethrin; a reduction in the number of muscle potential was already evident in the second 10 stimuli (Figure 5B). Similarly, upon exposure to deltamethrin, the number of muscle potentials was reduced in the fifth 10 stimuli in *w^1118^* flies. In *DSC1^a^* flies, the reduction in muscle potentials was detected in the second 10 stimuli and this reduction progressed in the remaining three groups of stimuli (Figure 5C). Thus, both type I and type II pyrethroids affect the GFS, and the *DSC1^a^* flies are more sensitive to pyrethroid-induced inhibition, as compared to *w^1118^* flies.

## Discussion

Four-domain, Ca^2+^-selective cation channels with a DEEA motif at the selectivity filter are interesting because their amino acid sequences and gating properties are intermediate between sodium and calcium channels. As such, these channels could potentially be an important evolutionary link between sodium and calcium channels. Although this family of cation channels has apparently been lost in vertebrates, they appear to be widespread in invertebrates. Despite the intriguing nature of the DEEA motif-containing cation channels, their role in animal physiology is not well understood. In this study, by taking advantage of the genetic tractability of *Drosophila melanogaster*, we assessed the role of DSC1, a prototype of DEEA motif-containing cation channels, in insect biology by generating null *DSC*1 knockout mutants using gene-targeted knockout via homologous recombination. Our behavioral, pharmacological and electrophysiological analyses of these mutants uncover an important role of the DSC1 channel in maintaining the overall stability of neural circuits, particularly under stressful environmental conditions.

Prior to our study, Anholt and colleagues [10] reported an olfactory phenotype in the *Drosophila smi60* line, which has reduced (by two-fold) *DSC1* transcript level associated with a *P*-element insertion in an intron. Our behavioral analysis of the *DSC1* knockout flies confirmed this pioneering observation and further shows that the knockout lines have a more severe olfaction defect compared to the *smi60* line. Besides the olfaction phenotype, however, we discovered that the *DSC1* knockout flies also exhibit a prominent jumpy phenotype when disturbed. In particular, this defect was intensified under heat shock and starvation conditions. Intriguingly, *DSC1* knockout flies are also more sensitive to pyrethroid insecticides.

What could be the common neural/physiological processes that directly or indirectly affect olfaction, jumpiness, and response to heat shock, starvation and pyrethroids? Our electrophysiological analyses using a well-defined neural circuit, the GFS, reveal that the DSC1 channel indeed contributes to nerve membrane excitability and may play an important role in balancing neuronal excitability and stability of synaptic transmission, thus providing an extended safety margin when the nervous system is required to operate beyond the normal functional range under challenging, extreme conditions.

First, we detected a defect in the refractory period of LL responses in the *DSC1* knockout flies upon heat shock and starvation. Consistent with earlier findings [21], heat shock reduced LLRP. We found that the reduction in the LLRP was more drastic in *DSC1* knockout flies than in *w^1118^* flies. The difference was most pronounced at 10 min of heat shock, at which the jumpy phenotype was also most distinct. Intriguingly, starvation also reduced LLRP in *DSC1* knockout flies (but not for *w^1118^*) and triggered the jumpy phenotype. Thus, reduced LLRP correlates with the jumpy phenotype, heat shock, and starvation. The LL response of the GF pathway is driven by sensory input from vision, olfaction and other sensory systems. In contrast, the SL response reflects direct activation of the GF and bypasses input from all the sensory circuits [22]. We found that deletion of the DSC1 channel did not cause severe alteration in the SL response of the DLMs (Figure S2) or its high-frequency responses (Figure 4B), suggesting that one of the key functions of the DSC1 channel is in modulating the activities of the central neurons presynaptic to the giant fiber. Deletion of the DSC1 channel enhanced the electrical activities of these neurons. This conclusion is consistent with the high level of DSC1 expression in the sensory system, such as the optic lobes, which send sensory input directly or indirectly to the GF [4].

Second, examination of the GF-TTM branch allows us to assess the synapses that connect the giant fiber to the motor neurons, and to the NMJ [22]. The synaptic defect appears to be in the GF terminal itself, not at the TTMn output since the response of TTMn was not altered when the TTM response was recruited by direct thorax stimulation. Furthermore, heat shock enhanced the following ability of the GF-TTM branch, again not the responses of DLMn and TTMn. These findings show that the DSC1 channel is not only involved in the frequency responses of GF at room temperature (Figure 4B), but also important for the regulation of GF function, possibly other neuronal elements in the nervous system when facing environmental challenges, such as heat shock (Figure 4C).

Third, destabilization of the GFS caused by the deletion of the DSC1 channel is also evident when depolarization at presynaptic neurons was intensified by the action of pyrethroids on sodium channels. Strikingly, both bioresmethrin (type I pyrethroid) and deltamethrin (type II pyrethroid) impaired the following ability of the SL response of the GFS during repetitive high voltage stimulation and this impairment was more severe in *DSC1* knockout flies, compared with *w^1118^* flies (Figure 5). The increased toxicity of pyrethroids on *DSC1* knockout flies is therefore likely because *DSC1* knockout flies have a hypersensitive nervous system.

Based on these findings, we propose that the DSC1 channel function as a stability guarding system to keep insect nerve firing properties in check, as evident by the greatly enhanced variability and deteriorated frequency response in the GF-TTM SL response (Figure 4B). This physiological function becomes even more apparent when the neural circuit is challenged under stressful conditions, such as heat shock, starvation or pyrethroid exposure (Figure 3, Figure 4, and Figure 5), which tend to hyper-stimulate the nervous system. How the DSC1/BSC1 cation channel exerts stability control over stress-enhanced neuronal hyperexcitability at the molecular level remains to be worked out. We have initiated such experiments in the larval neuromuscular preparation in which axonal action potentials and synaptic potentials can be directly recorded with microelectrodes. At present time, the preliminary results indicate defects in both axonal action potentials and neurotransmitter release. It is well known that synaptic transmission is regulated by calcium influxes. Calcium influx at the nerve terminals is mediated by voltage-gated calcium channels. Because the DSC1/BSC1 channel is a voltage-gated cation channel with relatively high permeability to Ca^2+^ [6], [7], it may have a fundamental role, together with classical voltage-gated calcium channels, in regulating Ca^2+^ fluxes at the nerve terminals. Intriguingly, unlike classical calcium channels, the DSC1 channel does not seem to play a critical role in regulating neurotransmitter release under normal laboratory condition since deletion of the DSC1 channel did not severely impair the functioning of the GFS under the normal laboratory condition. However, high-frequency stimulation, or heat shock, starvation or pyrethroid exposure, all of which induce excess neurotransmitter release [23], intensify the neural defects in *DSC1* knockout flies (Figure 3, Figure 4, Figure 5). The DSC1 channel could take part in the regulation of synaptic vesicle cycling, which is critical for synaptic transmission [24]. Both exocytotic release and re-uptake by endocytosis are controlled by Ca^2+^ influx [24], [25]. Interestingly, the identity of the calcium channel that controls endocytosis at the presynaptic terminals has not been identified in insects, raising the intriguing possibility that the DSC1/BSC1 channel could be a candidate. Conceivably, retarded cycling of synaptic vesicles at the presynaptic terminals, upon hyper-stimulation of nerve terminals in the absence of DSC1 channels, would almost certainly contribute to the defects in locomotion (jumpiness), and exasperated response to heat shock, starvation and pyrethroid exposure. It is also possible that deletion of the DSC1 channel somehow affect the expression or function of another ion channel, such as the sodium channel, that is important for maintaining the proper electric signaling in the nervous system. We examined the transcript level of the *para* sodium channel by quantitative-PCR, but did not detect a significant difference between *w^1118^* and *DSC1* knockout flies (Figure S3). Whether other ion channels in the nervous system are altered in the *DSC1* knockout flies to compensate for the loss of the DSC1 channel remains to be determined.

In summary, using *D. melanogaster DSC1* knockout mutants we have provided strong genetic evidence for an important role of the DSC1 channel in insect neurobiology and neurotoxicology. The results described in this study not only provide fundamental insight into the *in vivo* function of a prototypic member of an historically elusive family of ion channel, but also may have significant implications for the development of new and safer insecticides. In particular, the DSC1/BSC1-family cation channel, which are absent in vertebrates, may be excellent targets for the development of a new generation of pesticides. In addition, because the *DSC1* mutants are more susceptible to pyrethroids, DSC1 channel blockers may be useful to enhance the efficacy of pyrethroids, which are currently a key weapon against numerous agriculturally and medically important arthropod pests, including malaria-transmitting mosquitoes.

## Materials and Methods

### Plasmid construction

For constructing the donor transgene, a 6.6-kb *DSC1* genomic DNA region was amplified in two 3.3-kb fragments (Figure 1) by PCR (primer sequences are listed in Table S1) using genomic DNA isolated from *w^1118^* flies and platinum Taq DNA polymerase High Fidelity (Invitrogen). The upstream and downstream 3.3 kb fragments encode IS1–5, and IS6 and part of the linker connecting domain I and domain II, respectively. The DNA fragments were first cloned into PCR2.1 vector (Invitrogen). A stop codon along with a *Mlu*I (within ST1) or an *Eco*RI (within ST2) cleavage site was then introduced in the middle of the each fragment, (i.e., ST1 in the upstream fragment and ST2 in the downstream fragment, Figure 1), by PCR-mediated mutagenesis (primer sequences are listed in Table S1) using Pfu DNA polymerase (Stratagene). The PCR was carried out in a 50 µl reaction mixture for 18 cycles with 50 ng of DNA template and 2 units of Pfu DNA polymerase. The upstream fragment and the downstream fragments were then cloned into the pW25 vector (kindly provided by Dr. Kent G Golic, University of Utah) at the *Not*I and *Acc65*I sites, and the *BsiW*I and *Asc*I sites, respectively.

### Fly stocks and crosses

The gene knock-out strategy followed that reported by Rong and Golic [26]. Transgenic flies carry heat-inducible FLP recombinase (*70FLP*) and I-SceI endonuclease (*70I-SceI*) on chromosome 2 were kindly provided by Steve Crews (University of North Carolina, Chapel Hill). The donor constructs were transformed into *w^1118^* flies by standard P-element mediated transformation. The obtained transgenic flies that carry a donor construct on chromosome *X* or *3* were crossed with transgenic flies that carry both the heat-inducible *70FLP* and *70I-Sce*I transgenes. The 3–4 day old progeny were heat-shocked at 38°C for 1 h and crossed to *w* ^1118^ flies. When offspring with pigmented eyes were observed, the *w^+^* gene was mapped to detect its mobilization to chromosome *2* (to which *DSC1* maps).

### Southern blot analysis

Genomic DNA was isolated from 20–30 adult flies by standard methods. Southern blot analysis was performed using a DIG DNA Labeling and Detection Kit (Roche) according to the instruction manual. Briefly, DNA (∼10 µg) was cleaved with *Kpn*I, fractioned in a 0.8% agarose gel and transferred to nylon membrane (Amersham Pharmacia Biotech). Blots were probed with DIG-labeled DNA containing *DSC1* genomic DNA fragments (see Figure 1C). Hybridization was carried out for 16 h at 42°C in DIG Easy Hyb (Roche). Filters were washed twice for 15 min each at room temperature with 2× SSC containing 0.1% SDS, and twice for 30 min each at 68°C with 0.2× SSC containing 0.1% SDS.

### Heat shock assay

To avoid using damaged flies, a climbing assay (See Protocol S1) was performed and only flies that could climb into the top vial were collected for the heat shock assay. Flies (10 flies in each vial) were incubated in a 40°C humidified hybridization oven (Hybaid, Thermo Scientific, Inc.) with a glass front door which allowed direct observation of fly behavior during heat shock. The number of paralyzed flies was counted every minute during the 15-min heat shock period. Humidity in the chamber was maintained by including a plastic tray (8×12×3 cm) containing water. Paralysis is defined as loss of an ability to walk. In addition, the number of jumps in each vial was recorded from the 5th to 10th minutes during heat shock. A jump was defined as flying off from the wall or bottom of the vial.

### Recovery assay

Flies were returned to room temperature after the 15-min heat shock. A modified climbing assay was performed at various time points to determine recovery from the heat shock. The flies were tapped down to the bottom and given 30 seconds to stand up and climb. The number of flies that could climb on the wall of the vial was recorded. The assay was performed every 2 minutes during the first 20 minutes and every 5 minutes during the second 20 minutes of the recovery period.

### Starvation assay

The method was modified from a previous study [27]. Thirty two- to three-day-old male flies were evenly divided into six groups (i.e., five flies/vial) and raised on regular fly food for another two days before being transferred to vials containing 0.5% agar (five ml) instead of food. Flies were then transferred to fresh agar-containing vials daily and a jump phenotype similar to that induced by heat shock was examined daily for three days. The number of jumps per vial (five flies) was recorded during the first second to tenth seconds after a gentle tap on the vial.

### Insecticide bioassay

Deltamethrin, DCJW and fipronil were kindly provided by Bhupinder Khambay (Rothamsted Research, Ltd.), Keith Wing and Daniel Cordova (Dupont) and Vince Salgado (BASF), respectively. The method for contact bioassay was similar to that described in Hardstone et al., [28] (See Protocol S1 for details). The median lethal concentration (LC_50_) and 95% confidential interval were calculated using the POLOplus software (LeOra Software Company). LC_50_ values were considered as significantly different if the 95% confidence intervals did not overlap.

### Giant fiber system (GFS) recording

Methods for recording GF-driven muscle potentials with electrical stimulation similar to those used in Engel and Wu [11], [29], [30]. Briefly, the fly was tethered to a tungsten hook and electric stimuli (0.1 msec, Grass S88) were delivered across the brain through two tungsten electrodes inserted in the compound eyes just beneath the cornea (for long- and short-latency responses, anode in the right eye) or on the junction of prothorax and metathorax (for thorax stimulation of motor neurons, anode on the right side). The action potentials in the left tergotrochanteral muscle (TTM, leg extensor) and the right dorsal longitudinal muscle a (DLMa, indirect flight muscle) were recorded to indicate the giant fiber pathway output. Long and short latency response thresholds were assessed using single-pulse test stimuli with duration of 0.1 ms. Muscle responses were amplified by a DAM50 DC amplifier (World Precision Instrument, Inc.) and converted to digital signal by a Digidata 1440A (Axon Instrument, Inc.) coupled with Clampex 10.2 and Clampfit 10.2 software (Axon Instrument, Inc.).

### Statistics

Student's *t*-test was used to analyze the jump phenotype during heat shock and the climbing assay results. Two-way analysis of variance (ANOVA) with Tukey's test employed as the *post hoc* test were used to analyze the data of GFS recording. F-test was used to compare sample variances to detect differences in sample distribution. P<0.05 was set as the criterion for statistical significance.

## Supporting Information

Figure S1 “DART” assay (A. B.) and climbing assay (C.) of *w^1118^* and *DSC1* knockout flies. The response of *DSC1* flies to insect repellents benzaldehyde (A) and citronellal (B) is reduced compared with *w^1118^* flies. C. The climbing activity of *DSC1* knockout flies is comparable to that of *w^1118^* flies. (** *p*<0.001, * *p*<0.05, Student's *t*-test).
(TIF)

Figure S2 The latency and refractory period of the short latency pathway recorded during heat shock (A. B.), starvation (C. D.), and recovery from heat shock (E. F.). SL, short latency; SLRP, short latency refractory period; RT, room temperature. (* *p*<0.05, Two-way ANOVA).
(TIF)

Figure S3 *DSC1* knockout does not alter the level of *para* mRNA compared with *w^1118^*. Values given are means ± SEM (*n* = 3).
(TIF)

Protocol S1 Supporting Materials and Methods.
(DOCX)

Table S1 Primers used in this study.
(DOCX)

Table S2 ED_50_ of *w^1118^* and *DSC1^a^* flies.
(DOCX)

Table S3 Response latencies (ms) and refractory period of GFS of *w^1118^* and *DSC1^a^* flies measured at different time points of heat shock process (mean ± SD).
(DOCX)

Table S4 Response latencies and refractory periods of *w^1118^*, and *DSC1^a^* flies after 72 hours starvation (mean ± SD).
(DOCX)

Table S5 Response latencies (ms) and refractory period of GFS of *w^1118^* and *DSC1^a^* flies measured at different time points of recovery process (mean ± SD).
(DOCX)

Video S1 *DSC1* KO flies exhibit a jumpy phenotype at room temperature.
(AVI)

Video S2 *DSC1* KO flies are more jumpy than *w^1118^* flies after starvation.
(WMV)
