# Supplementary figures and images for: Role of the DSC1 Channel in Regulating Neuronal Excitability in Drosophila melanogaster: Extending Nervous System Stability under Stress
Source: PLoS Genet. 2013 Mar 7;9(3):e1003327. doi: 10.1371/journal.pgen.1003327 (PMC3591268; doi:10.1371/journal.pgen.1003327)

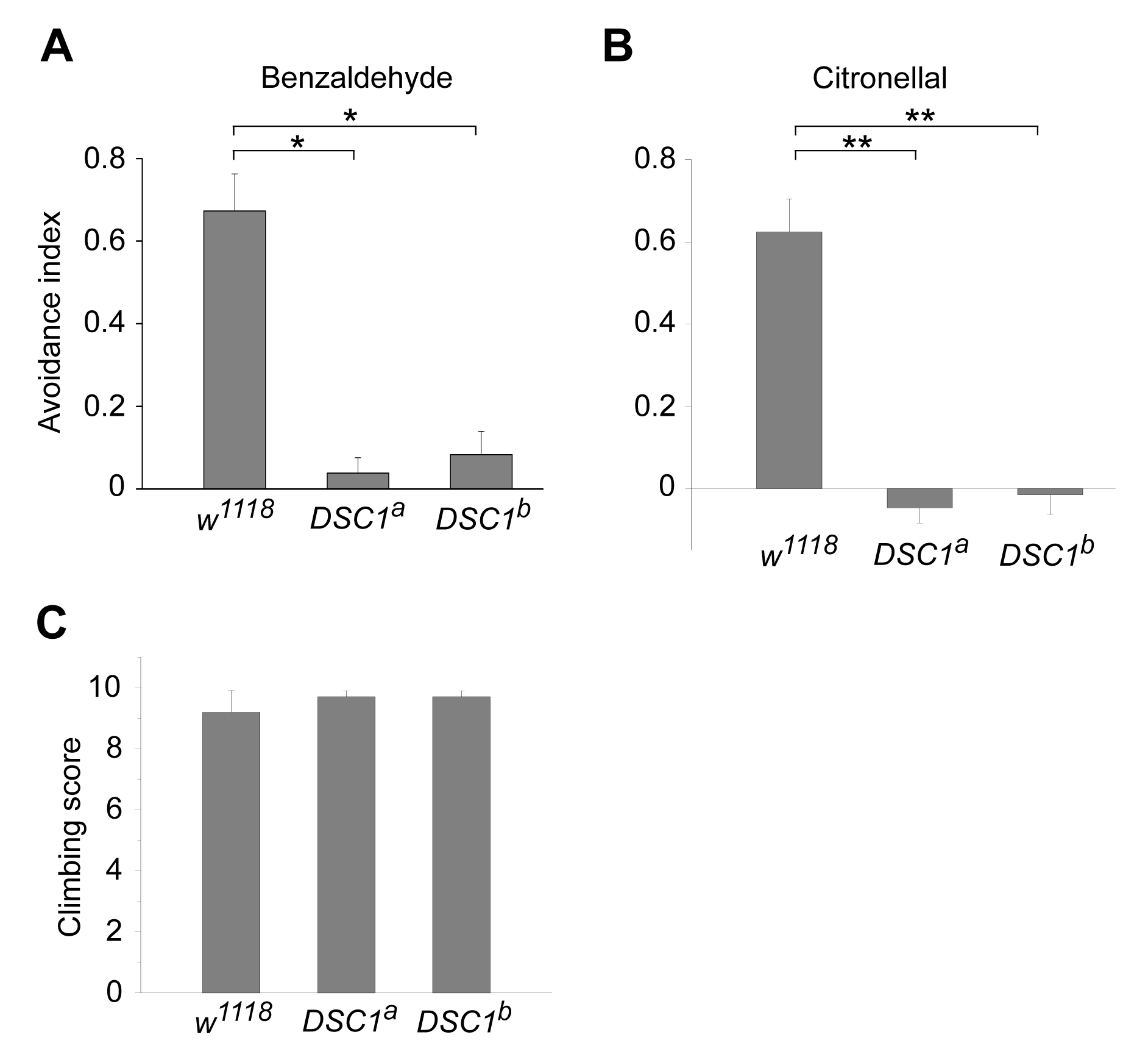

Supplement: Figure S1 — “DART” assay (A. B.) and climbing assay (C.) of w1118 and DSC1 knockout flies. The response of DSC1 flies to insect repellents benzaldehyde (A) and citronellal (B) is reduced compared with w1118 flies. C. The climbing activity of DSC1 knockout flies is comparable to that of w1118 flies. (** p<0.001, * p<0.05, Student's t-test). (TIF) [file pgen.1003327.s001.tif]

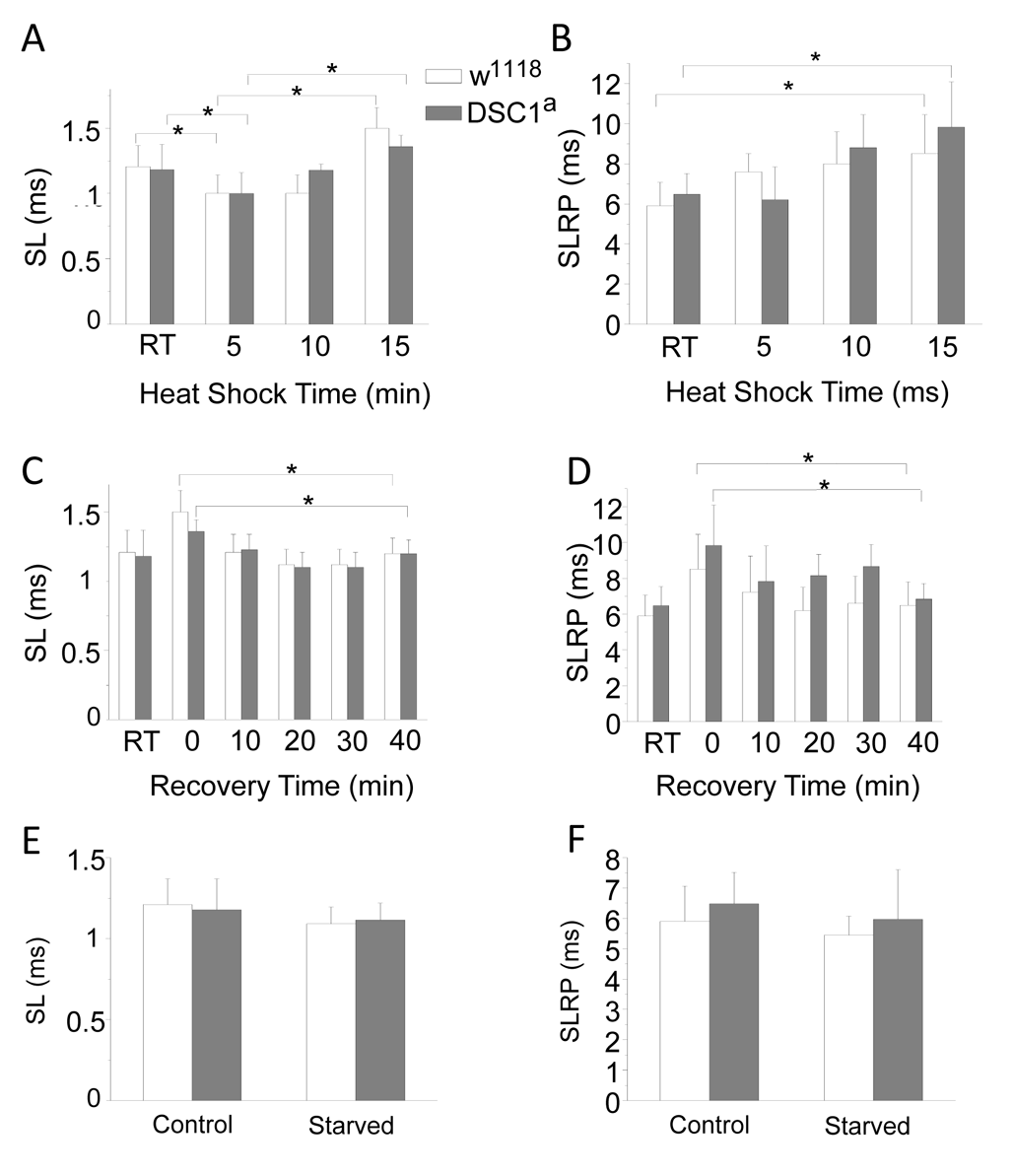

Supplement: Figure S2 — The latency and refractory period of the short latency pathway recorded during heat shock (A. B.), starvation (C. D.), and recovery from heat shock (E. F.). SL, short latency; SLRP, short latency refractory period; RT, room temperature. (* p<0.05, Two-way ANOVA). (TIF) [file pgen.1003327.s002.tif]

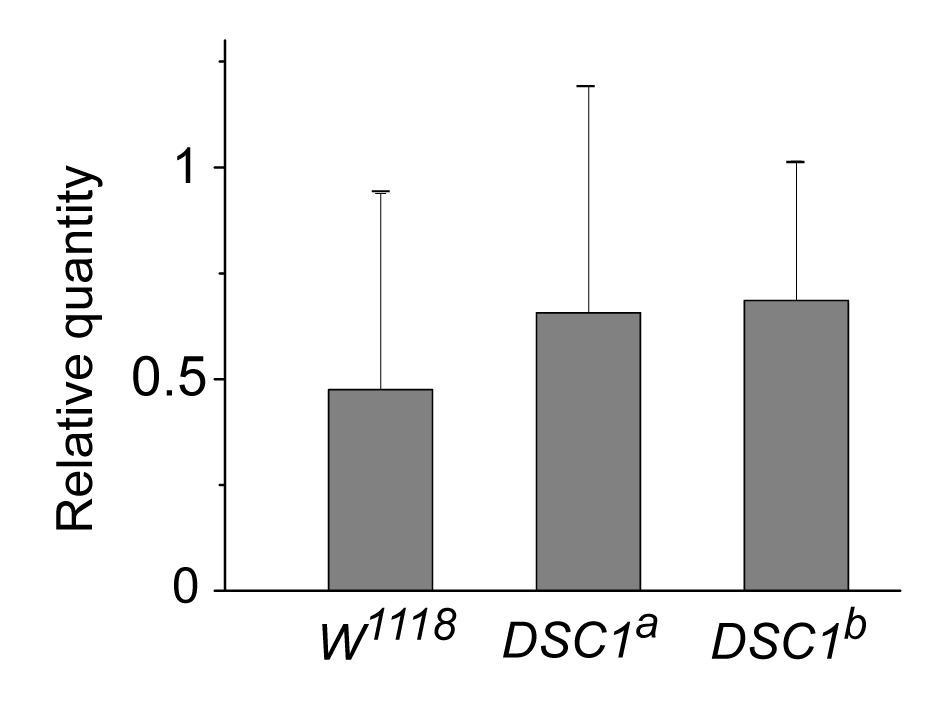

Supplement: Figure S3 — DSC1 knockout does not alter the level of para mRNA compared with w1118. Values given are means ± SEM (n = 3). (TIF) [file pgen.1003327.s003.tif]
