# Supplementary material for: Role of the DSC1 Channel in Regulating Neuronal Excitability in Drosophila melanogaster: Extending Nervous System Stability under Stress
Source: PLoS Genet. 2013 Mar 7;9(3):e1003327. doi: 10.1371/journal.pgen.1003327 (PMC3591268; doi:10.1371/journal.pgen.1003327)
Supplement: Protocol S1 — Supporting Materials and Methods. (DOCX) [file pgen.1003327.s004.docx]

**Supporting Materials and Methods**

**Climbing assay.** Five- to seven-day-old flies were collected and slightly immobilized by CO_2_. Ten flies (five males and five females) were transferred into a 95×25-mm plastic vial (Genesee Scientific). After the flies had acclimated for 30 minutes at room temperature, another empty vial was placed upside-down on top of the first vial. The rims of the two vials were aligned so that the flies could climb into the top vial freely. The bottom vials were tapped gently and files were given 30 seconds to climb. The number of the flies that climbed into the top vial was recorded and given a climbing score. For example, if there were eight flies that climbed into the top vial, the climbing score would be eight. This procedure was repeated five times for each group of flies and mean values were calculated for statistical analysis.

***Drosophila* “direct airborne repellent test (DART)” assays.** The chemicals used in this assay were citronellal and benzaldehyde (Sigma) diluted to 1% (v/v) using DMSO. For the DART assay, we followed the procedures described by Know et al. [1]. Briefly, a 5x5 mm portion of a Kimwipe was placed at the bottom of a 15ml test tube (Falcon, #352017, polystyrene). A total of 5 ul of diluted chemical was applied to the Kimwipe portion. A control tube was prepared in the same way but using DMSO only. A circular screen (Phifer) was placed ~5 mm from the bottom to prevent the flies from making direct physical contact with the chemical. 80-110 flies at age of 8-10 days old were used per chemical per assay. Testing zone definition and avoidance index calculation were exact same as described previously [1].

**Pyrethroid application.** 2- to 3-day-old male flies were slightly immobilized and mounted on a tungsten wire. Files were given 30 minutes to recover. Then, 0.4 ng deltamethrin (ED_95_ of knockdown for *w^1118^* flies, **Table S2**) or 4 ng/fly for bioresmethrin (bioresmethrin is 10-fold less potent than deltamethrin, **Table 1**) was topically delivered onto the dorsal side of thorax. For control group, only acetone was applied. The activities in the giant fiber system were recorded 15 min after pyrethroids exposure.

**Contact bioassay.** A 0.5 ml insecticide solution dissolved in acetone was delivered into a 25-ml glass scintillation vial. To coat the inner surface of the vial with insecticide, the vial was rolled on the side in a chemical fume hood for 3 to 5 min. Vials were kept in the hood for another 30 min to evaporate acetone. Twenty flies at the age of 2 to 3 days were slightly immobilized by CO_2_ and put into insecticide-coated scintillation vials. Vials were plugged by a cotton ball immersed with 3 ml of 20% sugar water. The vials were then kept at room temperature for 24 hours and the number of dead flies was counted at the end of assay. LD_50_ and 95% confidential interval were calculated using the POLOplus^TM^ software (LeOra Software Company, Petaluma, CA). The LD_50_ ratio is defined as the LD_50_ of *w^1118^* divided by the LD_50_ of *DSC1* knockout line.

**Quantitative Real-Time PCR.** Total RNA was extracted from 5 to 10 fresh adult fly specimens (3-7 days age) using WizardTM RNA isolation kit (Promega) as instructed in the user manual. The total amount of RNA was reverse transcribed using Superscript II reverse-transcriptase and oligo(dT)12-18 primer (Life Technologies) in a final volume of 20 μl. Quantitative real-time RT-PCR (qPCR) to determine the mRNA of para, the sodium channel gene in *Drosohila*, was performed on an Applied BiosystemsStepOnePlus Instrument and software (Applied Biosystems) using the DNA binding dye SYBERGreen I (Roche). The levels of *para* expression were normalized to actin using the ΔΔCt-method as specified by Applied Biosystems. To avoid genomic DNA contamination, PCR primers were designed to bind two fragments that are separated by an intron. PCR primers (dpara qf, dpara qr) are described in Table S1.

**Reference**

1. Kwon Y, Kim SH, Ronderos DS, Lee Y, Akitake B, et al. (2010) Drosophila TRPA1 channel is required to avoid the naturally occurring insect repellent citronellal. Curr Biol 20: 1672-1678.
