# Supplementary material for: Role of the DSC1 Channel in Regulating Neuronal Excitability in Drosophila melanogaster: Extending Nervous System Stability under Stress
Source: PLoS Genet. 2013 Mar 7;9(3):e1003327. doi: 10.1371/journal.pgen.1003327 (PMC3591268; doi:10.1371/journal.pgen.1003327)
Supplement: Table S1 — Primers used in this study. (DOCX) [file pgen.1003327.s005.docx]

**Table S1. Primers used in this study.**

| Name | Sequence |
| --- | --- |
| ST1 cloning forward primers | agcagcggccgcaacagaatatctccggttgcc |
| ST1 cloning reverse primers | taccggtaccagatttgacggactcacctc |
| ST2 cloning forward primers | agcaggcgcgcctaatggctctgcaggaacg |
| ST2 cloning reverse primers | tacccgtacgtcacctctctccagaccaac |
| ST1 mutagenesis forward primer | cgtcatggccaccattctgttc |
| ST1 mutagenesis reverse primer | cgttagtcgaagaactggttgg |
| ST2 mutagenesis forward primer | ttctgttaacacagcctttttggcc |
| ST2 mutagenesis reverse primer | ttcaaagtgtgatggccagctc |
| a | acacaaatgtgcaggcacagtgg |
| b | tacccgtacgtcacctctctccagaccaac |
| c | ctgggcgattcatagattcc |
| dpara qf | tcctgctctttcagatgtcg |
| dpara qr | tcattgataatggcgtccag |
| *Drosophila* actin forward primer | gagcgcggttacagcttca |
| *Drosophila* actin reverse primer | tccttgatgtcgcgcaca |
|  |  |
