# Supplementary material for: Role of the DSC1 Channel in Regulating Neuronal Excitability in Drosophila melanogaster: Extending Nervous System Stability under Stress
Source: PLoS Genet. 2013 Mar 7;9(3):e1003327. doi: 10.1371/journal.pgen.1003327 (PMC3591268; doi:10.1371/journal.pgen.1003327)
Supplement: Table S2 — ED50 of w1118 and DSC1a flies. (DOCX) [file pgen.1003327.s006.docx]

**Table S2**  **ED_50_ of *w^1118^* and *DSC1^a^* flies**

|  | ED_50_ (95% CI) (ng/fly) | ED_95_ (95% CI) (ng/fly) | *n* | Slope (SE) |
| --- | --- | --- | --- | --- |
| *w^1118^* | 0.067 (0.055-0.083) | 0.376 (0.262-0.638) | 240 | 2.20 (0.24) |
| *DSC1^a^* | 0.034 (0.026-0.044) | 0.298 (0.195-0.568) | 240 | 1.75 (0.21) |

We assessed the susceptibility of *w^1118^* and *DSC1* flies to knockdown 15 minutes after a series of doses of deltamethrin were delivered to the cuticle of dorsal thorax in a topical bioassay. ED_50_ is the dose that causes 50% knockdown of tested flies. These results confirmed that *DSC1^a^* flies were more vulnerable to deltamethrin than *w^1118^* flies.
