# Supplementary material for: Role of the DSC1 Channel in Regulating Neuronal Excitability in Drosophila melanogaster: Extending Nervous System Stability under Stress
Source: PLoS Genet. 2013 Mar 7;9(3):e1003327. doi: 10.1371/journal.pgen.1003327 (PMC3591268; doi:10.1371/journal.pgen.1003327)
Supplement: Table S3 — Response latencies (ms) and refractory period of GFS of w1118 and DSC1a flies measured at different time points of heat shock process (mean ± SD). (DOCX) [file pgen.1003327.s007.docx]

**Table S3. Response latencies (ms) and refractory period of GFS of *w^1118^* and *DSC1^a^* flies measured at different time points of heat shock process (mean ± SD)**

|  | | Heat Shock Time | | | |
| --- | --- | --- | --- | --- | --- |
|  | | RT | 5 min | 10 min | 15 min |
| SL | *w^1118^* | 1.2 ± 0.2 (n=17) | 1.0 ± 0.1 (n=5) | 1.0 ± 0.1 (n=5) | 1.5 ± 0.2 (n=10) |
|  | *DSC1^a^* | 1.2 ± 0.2 (n=17) | 1.0 ± 0.2 (n=5) | 1.2 ± 0.0 (n=5) | 1.4 ± 0.1 (n=10) |
| SLRP | *w^1118^* | 5.9 ± 1.2 (n=20) | 7.6 ± 0.9 (n=5) | 8.0 ± 1.6 (n=5) | 8.5 ± 1.9 (n=11) |
|  | *DSC1^a^* | 6.5 ± 1.0 (n=23) | 6.2 ± 1.6 (n=5) | 8.8 ± 1.6 (n=5) | 9.8 ± 2.3 (n=11) |
| LL | *w^1118^* | 3.8 ± 0.4 (n=22) | 3.8 ± 0.3 (n=7) | 3.9 ± 0.4 (n=9) | 3.5 ± 0.1 (n=7) |
|  | *DSC1^a^* | 3.8 ± 0.3 (n=24) | 3.6 ± 0.2 (n=5) | 3.9 ± 0.3 (n=8) | 3.4 ± 0.1 (n=11) |
| LLRP | *w^1118^* | 46.0 ± 9.3 (n=21) | 39.1 ± 5.2 (n=9) | 44.3 ± 8.0 (n=9) | 26.9 ± 7.0 (n=7) |
|  | *DSC1^a^* | 46.8 ± 15.9 (n=25) | 31.0 ± 4.8 (n=8) | 23.4 ± 2.8 (n=8) | 23.6 ± 3.1 (n=11) |
