# Supplementary material for: Role of the DSC1 Channel in Regulating Neuronal Excitability in Drosophila melanogaster: Extending Nervous System Stability under Stress
Source: PLoS Genet. 2013 Mar 7;9(3):e1003327. doi: 10.1371/journal.pgen.1003327 (PMC3591268; doi:10.1371/journal.pgen.1003327)
Supplement: Table S4 — Response latencies and refractory periods of w1118, and DSC1a flies after 72 hours starvation (mean ± SD). (DOCX) [file pgen.1003327.s008.docx]

**Table S4.** **Response latencies and refractory periods of *w^1118^*, and *DSC1^a^* flies after 72 hours starvation (mean ± SD)**

|  | SL (ms) | SLRP (ms) | LL (ms) | LLRP (ms) |
| --- | --- | --- | --- | --- |
| *w^1118^* | 1.1 ± 0.1 (n=13) | 5.5 ± 0.6 (n=13) | 3.5 ± 0.2 (n=13) | 49.2 ± 13.8 (n=13) |
| *DSC1^a^* | 1.1 ± 0.1 (n=12) | 6.0 ± 1.6 (n=12) | 3.3 ± 0.2 (n=12) | 35.3 ± 5.8 (n=12) |
