# Supplementary material for: Role of the DSC1 Channel in Regulating Neuronal Excitability in Drosophila melanogaster: Extending Nervous System Stability under Stress
Source: PLoS Genet. 2013 Mar 7;9(3):e1003327. doi: 10.1371/journal.pgen.1003327 (PMC3591268; doi:10.1371/journal.pgen.1003327)
Supplement: Table S5 — Response latencies (ms) and refractory period of GFS of w1118 and DSC1a flies measured at different time points of recovery process (mean ± SD). (DOCX) [file pgen.1003327.s009.docx]

**Table S5. Response latencies (ms) and refractory period of GFS of *w^1118^* and *DSC1^a^* flies measured at different time points of recovery process (mean ± SD)**

|  | | Recovery Time | | | |
| --- | --- | --- | --- | --- | --- |
|  | | 10 min | 20 min | 30 min | 40 min |
| SL | *w^1118^* | 1.2 ± 0.1 (n=12) | 1.1 ± 0.1 (n=5) | 1.1 ± 0.1 (n=5) | 1.2 ± 0.1 (n=9) |
|  | *DSC1^a^* | 1.2 ± 0.1 (n=10) | 1.1 ± 0.1 (n=6) | 1.1 ± 0.1 (n=6) | 1.2 ± 0.1 (n=11) |
| SLRP | *w^1118^* | 7.5 ± 2.1 (n=12) | 6.2 ± 1.3 (n=5) | 6.6 ± 1.5 (n=5) | 6.5 ± 1.3 (n=10) |
|  | *DSC1^a^* | 7.6 ± 1.9 (n=10) | 8.2 ± 1.2 (n=6) | 8.7 ± 1.2 (n=6) | 6.9 ± 0.9 (n=11) |
| LL | *w^1118^* | 4.0 ± 0.1 (n=12) | 4.1 ± 0.2 (n=11) | 3.9 ± 0.2 (n=6) | 3.9 ± 0.2 (n=7) |
|  | *DSC1^a^* | 3.8 ± 0.2 (n=9) | 3.9 ± 0.1 (n=8) | 3.6 ± 0.2 (n=8) | 3.7 ± 0.2 (n=9) |
| LLRP | *w^1118^* | 44.6 ± 7.8 (n=12) | 48.7 ± 8.6 (n=11) | 44.8 ± 6.0  (n=6) | 49.7 ± 10.0 (n=7) |
|  | *DSC1^a^* | 26.3 ± 2.1 (n=9) | 32.6 ± 5.6 (n=8) | 30.1 ± 4.3 (n=8) | 31.4 ± 4.8 (n=9) |
